# Morphological changes in the palate after transverse expansion with removable orthodontic plate appliances: Short-term effects and follow-up stability

**DOI:** 10.1007/s00056-025-00592-z

**Published:** 2025-06-11

**Authors:** Gero Stefan Michael Kinzinger, Jan Hourfar, Hee Jung Kim, Jörg Alexander Lisson

**Affiliations:** Department of Orthodontics, Saarland University, Homburg, Germany

**Keywords:** Maxillary constriction, Removable orthodontic appliances, Cast analysis, Dentoalveolar effects, Sutures, Gaumenkonstriktion, Herausnehmbare kieferorthopädische Apparaturen, Modellanalyse, Dentoalveoläre Effekte, Suturen

## Abstract

**Background and aim:**

The three-dimensional effects of fixed rapid maxillary expansion (RME) on palatal morphology for the treatment of maxillary constriction have been extensively studied. Morphological changes caused by treatment with removable plate appliances have not been investigated. Therefore, the aim of this retrospective cohort study was to analyse changes and follow-up stability in palatal width, height and surface area after transverse expansion with removable plate appliances in patients of different age groups.

**Patients and methods:**

The course of treatment of *n* = 90 children and adolescents, documented through dental casts, was quantified using various analyses. The patient cohort (PC) was divided into three groups according to chronological age for analysis: PG 1 < 10 years of age (*n* = 30, early treatment), PG 2 10 to < 13 years of age (*n* = 30, main treatment) and PG 3 ≥ 13 years of age (*n* = 30, late treatment). Data were collected before treatment (T1), after transverse expansion of the palate (T2) and after completion of or retention after orthodontic treatment (T3).

**Results:**

The average treatment interval (T1–T2) was 16.8 months. The average observation period (T2–T3) was 20.2 months. Maxillary expansion with the plate appliances occurred evenly regardless of patient age, with the greatest effects in the posterior molar region. The expansion remained stable until T3 regardless of further measures. The therapeutically induced increases in width, height and area were not significantly different between the groups despite lower initial values in the younger patients.

**Conclusions:**

Compared to treatment with fixed RME appliances, where the effects differ due to age-related sutural changes, the morphological changes with plate appliances are comparable in patients of different chronological age. Early, main and late treatment were equally effective. This may be because the changes after treatment with plate appliances occur mainly through dentoalveolar effects, while effects on the sutures play, if any, only a minor role.

## Abstract (de)

**Hintergrund und Ziel:**

Dreidimensionale Effekte der festsitzenden Gaumennahterweiterung (RME) auf die Gaumenmorphologie zur Behandlung von Konstriktionen des Oberkiefers wurden umfangreich wissenschaftlich evaluiert. Vergleichbare morphologische Veränderungen mit herausnehmbaren Plattenapparaturen als therapeutische Alternative sind bislang nicht untersucht worden. Ziel der vorliegenden Studie war deshalb zunächst, an Patienten unterschiedlicher Altersgruppen durch Modellanalyse Veränderungen der Gaumenbreite, -höhe und -fläche nach transversaler Erweiterung mit Plattenapparaturen zu analysieren. Anschließend sollte die Langzeitstabilität der Veränderungen evaluiert werden.

**Patienten und Methoden:**

Die Behandlungsverläufe von 90 Kindern und Jugendlichen wurden durch Modelle dokumentiert und anhand von verschiedenen Analysen quantifiziert. Nach chronologischem Alter konnte in 3 zahlenmäßig gleich große Patientengruppen (PG) unterteilt werden (PG1: Patienten bis zum 10. Lebensjahr, *n* = 30; PG2: Patienten vom 10.–12. Lebensjahr, *n* = 30; PG3: Patienten ab dem 12. Lebensjahr, *n* = 30). Die Daten wurden vor der Behandlung (T1), nach der transversalen Erweiterung des Gaumens (T2) und nach Abschluss bzw. Retention der kieferorthopädischen Behandlung (T3) erhoben.

**Ergebnisse:**

Das durchschnittliche Behandlungsintervall (T1–T2) betrug 16,8 Monate. Der durchschnittliche Beobachtungszeitraum (T2–T3) betrug 20,2 Monate. Die maxilläre Erweiterung durch Plattenapparaturen erfolgt unabhängig vom Patientenalter weitestgehend gleichförmig mit den größten Effekten im posterioren Molarenbereich und bleibt im Beobachtungszeitraum unabhängig von weiteren Maßnahmen stabil. Die therapeutisch bedingten Zunahmen von Breite, Höhe und Fläche unterscheiden sich zwischen den Gruppen trotz anatomisch bedingt geringeren Ausgangswerten bei den jüngeren Patienten nur unwesentlich.

**Schlussfolgerungen:**

Anders als bei der Behandlung mit festsitzenden RME-Apparaturen, bei denen die Effekte aufgrund altersbedingter suturaler Veränderungen unterschiedlich ausfallen, sind die morphologischen Veränderungen mit Plattenapparaturen bei Patienten unterschiedlichen Alters vergleichbar. Früh‑, Haupt- und Spätbehandlungen waren gleichermaßen effizient. Dies mag daran liegen, dass die Behandlungseffekte mit Plattenapparaturen überwiegend auf dentoalveoläre Effekte zurückzuführen sind und Auswirkungen auf die Suturen nur eine untergeordnete Rolle spielen.

## Introduction

Constriction of the maxilla is a common anomaly. The causes of a narrow palate are multicausal [1]. Several determinants have been discussed: in addition to genetic causes [2], environmental factors such as different standards of living and the associated differences in diet [3, 4] or the prolonged use of a pacifier are considered risk factors for the development of a narrow jaw and reduced dental arch width [5, 6]. In addition, a habitual deviation from the somatic to the visceral swallowing pattern, in which the tongue is pushed forward instead of resting against the palate, can lead to a narrow jaw [7, 8]. Arnar et al. [5] also cite mouth breathing as a risk factor for a narrow dental arch. Lione et al. [9] showed that impaired oral breathing due to allergic rhinitis resulted in elevation of the palate and narrowing of the dental arch in 8 year olds compared with healthy individuals.

The correction of an excessively narrow upper jaw can be achieved with either fixed or removable orthodontic appliances. The morphological changes in the tooth-bearing palate are of particular interest. A study group of Kinzinger et al. was the first to carry out extensive dental cast studies on the effects of the fixed rapid maxillary expansion (RME) [10–12]. Using a combined cone-beam computed tomography (CBCT) and cast study, they demonstrated that the therapeutic effects of RME on palatal morphology are age dependent [11]. They concluded that maxillary expansion after RME tends to be uniform in children up to 10 years of age, whereas with increasing age—especially in adolescents from 12 years of age—it becomes V‑shaped (anterior > posterior transverse, inferior > superior vertical). In addition to age-related rigidity of the pterygopalatomaxillary sutures, this appears to be due to morphological changes in the transverse palatine suture during growth. Thus, age-dependent effects of rapid palatal expansion are based on a change in the position of the maxillary centers of rotation and resistance. It remains questionable whether there are age-related differences in the therapeutic effects of maxillary transverse expansion with removable plate appliances, which have no direct sutural effects.

## Aim

The present retrospective cohort study intends to provide answers to the following questions:

- What short- and long-term morphological changes in the maxilla and the dental arch can be expected through slow transverse expansion with plate appliances?
- Do width, height and surface of the tooth-bearing palate change differently in the anterior and posterior regions of the maxilla after plate appliance treatment at different patient ages?
- Is early treatment prognostically more favorable and late treatment still effective?

## Materials and methods

### Patients

The study examined nonrandomized participants. Subjects were enrolled based on strict criteria for the removable plate appliances used in the treatment. These were always constructed with a transverse screw as the only active element for tooth movement.

**Fig. 1:**
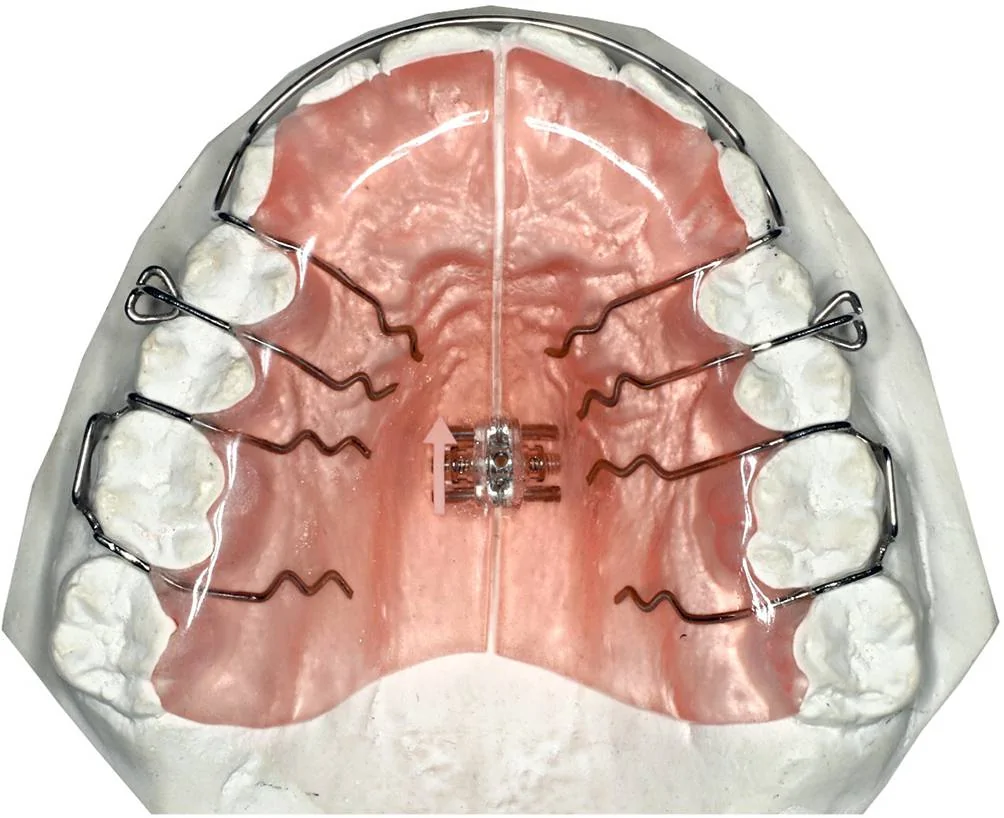
Maxillary plate appliance with expansion screw Oberkiefer-Plattenapparatur mit Transversalschraube

The study group consisted of 90 patients (46 female, 44 male), who were treated between August 2013 and November 2022 with plate appliances designed exclusively for transverse expansion of the maxillary dental arch (Fig. 1).

The study subjects were divided into three patient groups (PG) according to age: PG1 up to the age of 10 years, PG2 between 10 and 12 years and PG3 above the age of 12. Assignment to the respective PG referred to the age at which the orthodontic appliance was inserted. In all, 30 patients from each age group were included in the study.

Inclusion criteria were constricted upper dental arch with resulting crossbite (right, left or both sides) due to dental arch width discrepancies to the lower jaw. Patients with previous orthodontic treatment, extractions, missing teeth or missing teeth in the support zones due to tooth replacement were excluded.

The actual number of screw activations and the extent of any resetting of the screw spindles as well as the exact wearing time of the plate appliances were not checked or documented.

### Data acquisition

Data were collected at three defined time points: T1 before treatment, T2 after transverse expansion of the palate with the plate appliance, and T3 after completion of or retention after orthodontic treatment. Between T2 and T3, some of the patients were treated with functional orthodontic appliances and some with comprehensive fixed appliances without further active transverse expansion. The effects between T1 and T2 or T2 and T3 are short-term effects, whereas the time interval between T1 and T3 reflects the total treatment effect.

### Orthodontic appliances

The orthodontic treatment between time points T1 and T2 was carried out with removable plate appliances (Fig. 1) containing a transverse screw (article no. 100–2000, Forestadent, Pforzheim, Germany) for expansion. The expansion was achieved by regularly activating the screw spindle (instructions: one activation per week, lift height 0.8 mm, 0.2 mm per activation) until the therapeutic goal (crossbite alignment, congruence of the maxillary and mandibular arches) was achieved. The prescribed wearing time was 12–14 h/day. After T2, the results were retained with either passive plate appliances or—in the case of continuing treatment—stabilization of the achieved results through comprehensive appliances. There was no further transverse expansion.

### Cast analysis

**Fig. 2:**
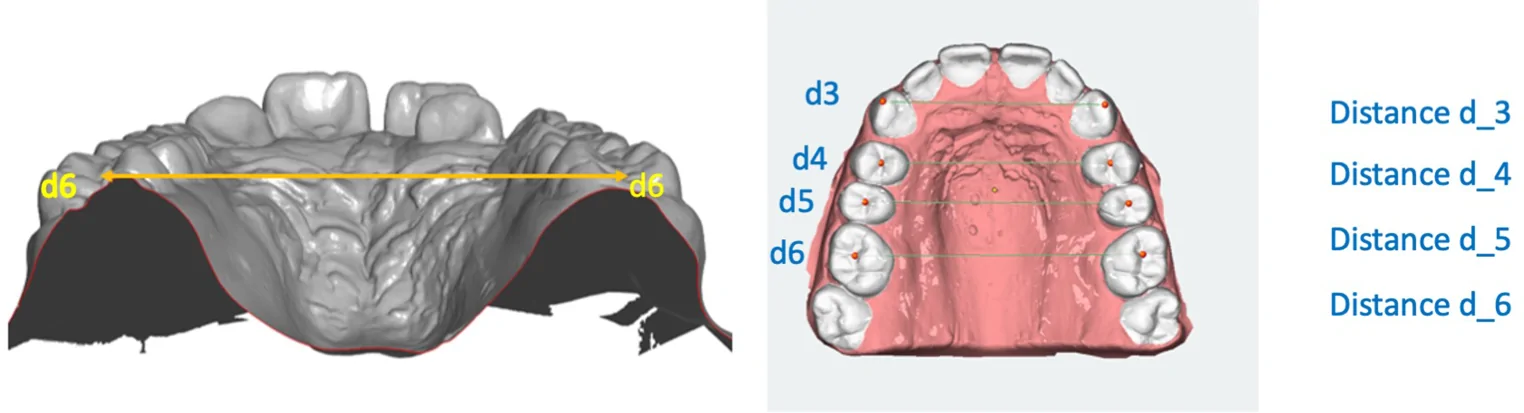
Measurement of the dental width on a digital model of the maxilla Vermessung der dentalen Breite an einem digitalen Oberkiefermodell

**Fig. 3:**
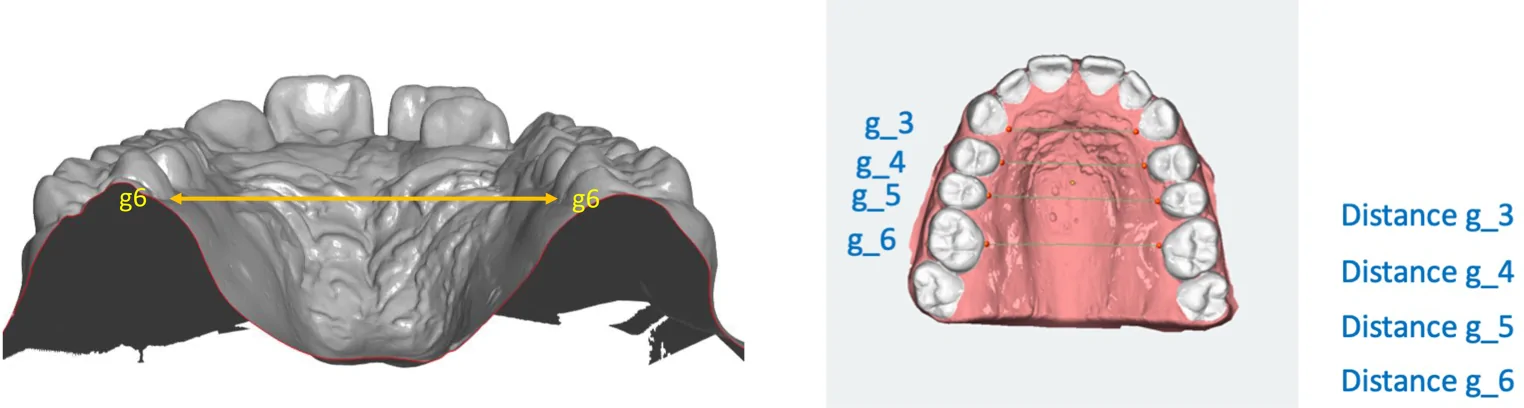
Measurement of the gingival width on a digital model of the maxilla Vermessung der gingivalen Breite an einem digitalen Oberkiefermodell

**Fig. 4:**
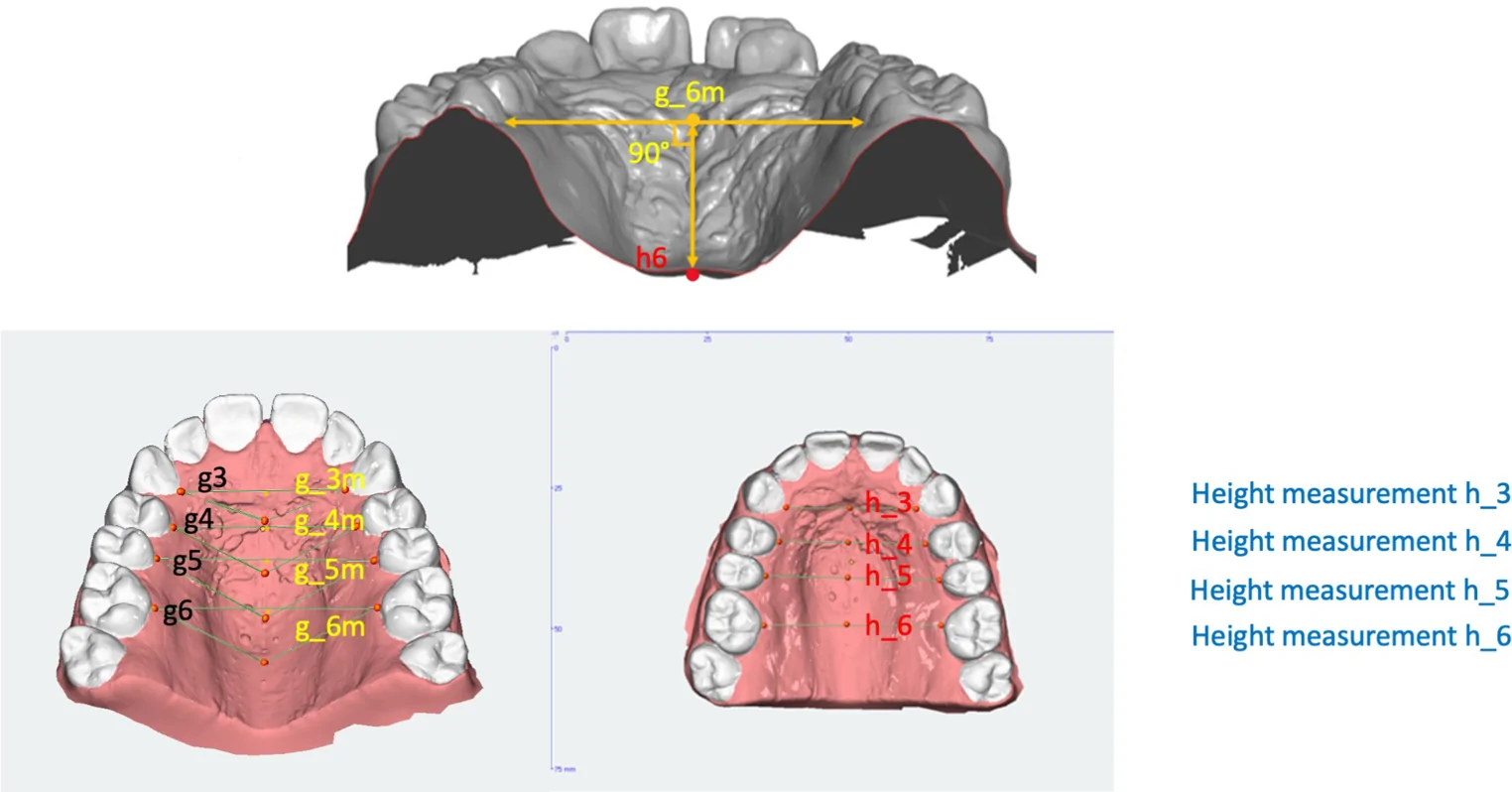
Measurement of the palatal height on a digital model of the maxilla Vermessung der Gaumenhöhe an einem digitalen Oberkiefermodell

**Fig. 5:**
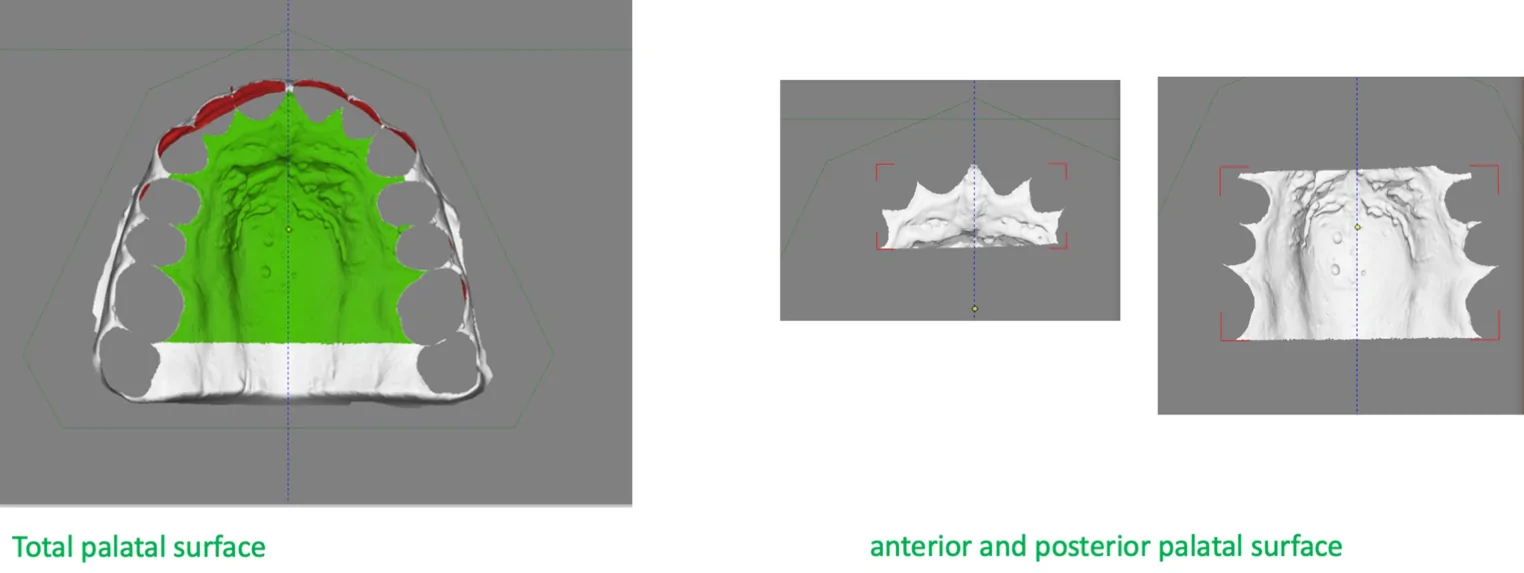
Measurement of the palatal surface (green total); anterior and posterior part Vermessung der Gaumenfläche (grün gesamt); anteriorer und posteriorer Teil

(Table 1, Figs. 2, 3, 4 and 5)

**Table 1:** Measuring points, distances and areas. Tooth numbers refer to the common FDI (Fédération Dentaire Internationale) scheme^a^ Messpunkte, -strecken und -flächen. Zahnzahlen beziehen sich auf das gängige FDI(Fédération Dentaire Internationale)-Zahnschema^a^

d3	Canine tip
d4	Central fissure of first deciduous molar/premolar
d5	Central fissure of second deciduous molar/premolar
d6	Central fissure of first permanent molar
d_3	Distance between left and right d3
d_4	Distance between left and right d4
d_5	Distance between left and right d5
d_6	Distance between left and right d6
g3	Lowest point on the gingival margin of the canine
g4	Lowest point on the gingival margin of the first deciduous molar/premolar
g5	Lowest point on the gingival margin of the second deciduous molar/premolar
g6	Lowest point on the gingival margin of the first permanent molar
g_3	Distance between left and right g3
g_4	Distance between left and right g4
g_5	Distance between left and right g5
g_6	Distance between left and right g6
h3	Centre of palate on median raphe line in area of the canines
h4	Centre of palate on median raphe line in area of the first deciduous molar/premolar
h5	Centre of palate on median raphe line in area of the second deciduous molar/premolar
h6	Centre of palate on median raphe line in area of the first permanent molar
g_3m	Intersection of h3 perpendicular to g_3
g_4m	Intersection of h3 perpendicular to g_4
g_5m	Intersection of h3 perpendicular to g_5
g_6m	Intersection of h3 perpendicular to g_6
h_3	Distance between h3 and g_3m
h_4	Distance between h4 and g_4m
h_5	Distance between h5 and g_5m
h_6	Distance between h6 and g_6.m
TPS	Total palatal surface
APS	Anterior palatal surface
PPS	Posterior palatal surface

^a^*3* canine, *4* first premolar/1st deciduous molar; *5* 2nd premolar/2nd deciduous molar, *6* 1st permanent molar

A total of 270 maxillary casts made at T1, T2 and T3 were analysed. The 3Shape TRIOS (3Shape, Copenhagen, Denmark) was used for digitizing the casts. The scanned casts were then imported and measured using the three-dimensional diagnostic software OnyxCeph3™Lab (Image Instruments GmbH, Chemnitz, Germany).

The dental arch width was measured from the canines to the first permanent molars at the measuring points according to Pont [13]. The width of the palate was also measured between the most coronal points of the gingival margin from the canine through the deciduous and premolars to the first permanent molars (gingivoalveolar plane).

On both vertical planes (dental, gingivoalveolar), the ratio of the anterior/posterior width between the first deciduous or premolars and the first molars was determined to assess the transverse expansion quality (values < 1 = inverse V‑shaped/delta-shaped; 0 = parallel, > 1 = V-shaped/triangular).

The height of the palate was determined by measuring the vertical connection of the median raphe line with the connecting lines of the most coronal points of the gingival margin on the canines, first and second premolars or deciduous molars, and first permanent molars. The anterior/posterior height ratio was calculated between the first deciduous or premolar and the first permanent molar (values < 1 = relatively greater posterior height increase; 0 = equal change in anterior and posterior height, > 1 = relatively greater anterior height increase). The width and height ratios indicate changes of the palatal depth in the sagittal plane.

The surface measurements were conducted according to Gracco et al. [14]. The dental cast was digitally sectioned to include only the soft tissue area. The dorsal plane was oriented perpendicular to the gingival plane and extended through the two most distal points of the first permanent molars. The entire palatal surface was then digitally measured. Afterwards, the anterior part was cut out by setting the reference point 3. RFP (intersection of the plane through the third pair of palatal folds and the median) and the inserted line parallel to the posterior connecting lines and measured digitally. The area of the posterior part was calculated by subtracting the anterior area from the total area, and the result was given in mm^2^.

### Statistics, error of the method

Anonymized patient data were collected using a spread sheet software (Excel®, Microsoft Corp., Redmond, WA, USA). Normal distribution of all variables was evaluated with the Shapiro–Wilk test with SPSS® version 30 for Windows® (IBM Corp., Armonk, NY, USA). Mean and standard deviation (SD) as well as confidence interval were reported for each variable.

Treatment-associated changes (∆T) in variables were analysed for intragroup comparisons using the paired t‑test. Differences between the groups were assessed with the analysis of variance (ANOVA). Post hoc testing was performed using the Tukey test. Homogeneity of variance was confirmed through the Levene test. Mean and standard deviation as well as confidence interval were reported for each variable. Statistical significance was assumed for *p*-values < 0.05.

The combined method error (ME) was determined according to Dahlberg [15]: 25% of the casts were randomly selected after a period of 3 months and measured again by the same investigator. The method error for all measurements was calculated with the formula ME = √(∑d^2^/2*n*) to determine the validity of the measurement method, where d is the difference between two measurement results and *n* is the number of duplicate measurements. The ME in the present study was < 1 for all measurements.

## Results

(Table 2, 3, 4, 5 and 6)

**Table 2:** Characteristics of the study group (*n* = 90) and respective patient groups (PG) 1–3 (*n* = 30 each) Charakterisierung der Gesamtstudiengruppe (*n* = 90) und der Patientengruppen (PG) 1–3 (jeweils *n* = 30)

	Total M	Total SD	PG1 M	PG1 SD	PG2 M	PG2 SD	PG3 M	PG3 SD
Insertion age (years)	10.7	2.2	8.1	0.9	10.9	0.6	13.0	0.9
∆ T1–T2 (months)	16.8	7.4	17.0	8.4	16.8	7.0	16.6	7.0
∆ T2–T3 (months)	20.2	9.8	19.5	14.3	20.8	9.5	20.5	10.6

*M* mean value; *SD* standard deviation; *T1* before treatment; *T2* after transverse palatal expansion; *T3* after completion of treatment

**Table 3:** Widths (dental and gingival), heights and areas (anterior, posterior and total) at T1, T2 and T3 in patient group 1, significance *p* intra Breiten (dental und gingival), Höhen und Flächen (anterior, posterior und gesamt) zu den 3 Zeitpunkten T1, T2 und T3 in der Patientengruppe 1, Signifikanzniveau *p* intra

PG1	T1	T2	T3	∆T2–T1		∆T3–T2		∆T3–T1	
Measurement	M ± SD 95% CI (LB, UB)	M ± SD 95% CI (LB, UB)	M ± SD 95% CI (LB, UB)	M ± SD 95% CI (LB, UB)	*p* (intra)	M ± SD 95% CI (LB, UB)	*p* (intra)	M ± SD 95% CI (LB, UB)	*p* (intra)
d_3	30.66 ± 2.04 29.90, 31.42	33.66 ± 2.29 32.81, 34.51	33.68 ± 2.27 32.84, 34.53	3.00 ± 2.11 2.21, 3.79	< 0.001 ***	0.02 ± 2.03 −0.73, 0.78	1.000 ^NS^	3.02 ± 1.98 2.28, 3.76	< 0.001 ***
d_4	33.35 ± 2.20 32.53, 34.17	36.59 ± 2.47 35.66, 37.51	36.15 ± 2.63 35.17, 37.13	3.23 ± 2.08 2.46, 4.01	< 0.001 ***	−0.44 ± 1.78 −1.10, 0.23	0.567 ^NS^	2.80 ± 1.96 2.06, 3.53	< 0.001 ***
d_5	38.90 ± 2.38 38.02, 39.79	42.87 ± 2.91 41.78, 43.95	42.51 ± 2.71 41.50, 43.53	3.96 ± 2.45 3.05, 4.88	< 0.001 ***	−0.35 ± 2.24 −1.19, 0.48	1.000 ^NS^	3.61 ± 2.22 2.78, 4.44	< 0.001 ***
d_6	44.05 ± 2.79 43.00, 45.09	48.11 ± 3.40 46.84, 49.38	47.76 ± 3.33 46.51, 49.01	4.07 ± 2.88 2.99, 5.14	< 0.001 ***	−0.35 ± 2.04 −1.11, 0.41	1.000 ^NS^	3.71 ± 2.60 2.74, 4.68	< 0.001 ***
Ratio d_4/d_6	0.76 ± 0.03 0.75, 0.77	0.76 ± 0.03 0.75, 0.77	0.76 ± 0.04 0.74, 0.77	0.00 ± 0.02 0.00, 0.01	1.000 ^NS^	0.00 ± 0.02 −0.01, 0.00	0.972 ^NS^	0.00 ± 0.02 −0.01, 0.01	1.000 ^NS^
g_3	23.48 ± 2.04 22.72, 24.24	25.90 ± 2.03 25.14, 26.65	25.53 ± 1.94 24.80, 26.25	2.42 ± 1.67 1.80, 3.04	< 0.001 ***	−0.37 ± 1.51 −0.93, 0.19	0.572 ^NS^	2.05 ± 1.88 1.35, 2.75	< 0.001 ***
g_4	25.61 ± 2.16 24.80, 26.41	28.50 ± 2.31 27.64, 29.37	27.69 ± 2.43 26.78, 28.60	2.90 ± 2.20 2.07, 3.72	< 0.001 ***	−0.81 ± 1.78 −1.48, −0.15	0.050 ^NS^	2.08 ± 2.05 1.32, 2.85	< 0.001 ***
g_5	28.25 ± 2.35 27.37, 29.13	31.99 ± 2.51 31.05, 32.93	32.20 ± 2.81 31.15, 33.25	3.74 ± 2.08 2.96, 4.52	< 0.001 ***	0.21 ± 2.18 −0.61, 1.02	1.000 ^NS^	3.95 ± 2.19 3.13, 4.76	< 0.001 ***
g_6	31.06 ± 2.59 30.09, 32.02	34.65 ± 2.83 33.59, 35.71	34.32 ± 2.98 33.20, 35.43	3.59 ± 2.46 2.68, 4.51	< 0.001 ***	−0.33 ± 2.02 −1.09, 0.42	1.000 ^NS^	3.26 ± 2.28 2.41, 4.11	< 0.001 ***
Ratio g_4/g_6	0.83 ± 0.05 0.81, 0.84	0.82 ± 0.05 0.81, 0.84	0.81 ± 0.05 0.79, 0.83	0.00 ± 0.03 −0.01, 0.01	1.000 ^NS^	−0.02 ± 0.04 −0.03, 0.00	0.118 ^NS^	−0.02 ± 0.04 −0.03, 0.00	0.037 *
h_3	3.77 ± 1.30 3.28, 4.26	4.28 ± 1.51 3.72, 4.84	4.15 ± 1.45 3.61, 4.69	0.51 ± 0.87 0.18, 0.84	0.010 *	−0.13 ± 0.85 −0.45, 0.18	1.000 ^NS^	0.38 ± 0.90 0.04, 0.71	0.086 ^NS^
h_4	8.95 ± 1.58 8.36, 9.54	9.63 ± 1.69 9.00, 10.26	9.49 ± 1.84 8.80, 10.18	0.68 ± 0.82 0.37, 0.99	< 0.001 ***	−0.14 ± 1.06 −0.54, 0.26	1.000 ^NS^	0.54 ± 1.16 0.11, 0.97	0.050 ^NS^
h_5	11.62 ± 1.64 11.00, 12.23	12.41 ± 1.86 11.71, 13.10	13.04 ± 1.95 12.31, 13.76	0.79 ± 1.14 0.36, 1.22	0.002 **	0.63 ± 1.29 0.15, 1.11	0.037 *	1.42 ± 1.12 1.00, 1.84	< 0.001 ***
h_6	10.44 ± 1.69 9.81, 11.07	11.39 ± 2.08 10.61, 12.17	12.68 ± 2.79 11.64, 13.72	0.95 ± 1.30 0.47, 1.43	0.001 **	1.29 ± 1.39 0.77, 1.81	< 0.001 ***	2.24 ± 1.84 1.55, 2.93	< 0.001 ***
Ratio h_4/h_6	0.87 ± 0.17 0.81, 0.94	0.76 ± 0.03 0.75, 0.77	0.77 ± 0.17 0.71, 0.83	−0.01 ± 0.12 −0.05, 0.04	1.000 ^NS^	−0.10 ± 0.10 −0.14, −0.06	< 0.001 ***	−0.10 ± 0.13 −0.15, −0.05	< 0.001 ***
APS	342.76 ± 62.32 319.49, 366.03	386.73 ± 64.23 362.74, 410.71	386.53 ± 63.34 362.88, 410.18	43.97 ± 39.24 29.31, 58.62	< 0.001 ***	−0.20 ± 36.99 −14.01, 13.61	1.000 ^NS^	43.77 ± 45.86 26.64, 60.89	< 0.001 ***
PPS	969.56 ± 131.45 920.48, 1018.65	1045.45 ± 142.71 992.16, 1098.73	1040.57 ± 156.06 982.30, 1098.85	75.88 ± 67.13 50.82, 100.95	< 0.001***	−4.87 ± 56.69 −26.04, 16.30	1.000 ^NS^	71.01 ± 83.19 39.95, 102.07	< 0.001 ***
TPS	1312.32 ± 160.42 1252.42, 1372.23	1432.17 ± 172.61 1367.72, 1496.63	1427.10 ± 195.59 1354.06, 1500.13	119.85 ± 88.04 86.98, 152.72	< 0.001 ***	−5.08 ± 73.62 −32.57, 22.42	1.000 ^NS^	114.77 ± 108.73 74.17, 155.38	< 0.001 ***

*M* mean, *SD* standard deviation, *CI* confidence intervals and significance levels, *LB* lower bound, *UB* upper bound, *NS* not significant, *PG* patient group, *APS* anterior palatal surface, *PPS* posterior palatal surface, *TPS* total palatal surface

* *p* < 0.05, ** *p* < 0.01, *** *p* < 0.001

Distance measurements in mm, area measurements in mm^2^

**Table 4:** Widths (dental and gingival), heights and areas (anterior, posterior and total) at T1, T2 and T3 in patient group (PG) 2, significance *p* intra Breiten (dental und gingival), Höhen und Flächen (anterior, posterior und gesamt) zu den 3 Zeitpunkten T1, T2 und T3 in der Patientengruppe 2, Signifikanzniveau *p* intra

PG2	T1	T2	T3	∆T2–T1		∆T3–T2		∆T3–T1	
Measurement	M ± SD 95% CI (LB, UB)	M ± SD 95% CI (LB, UB)	M ± SD 95% CI (LB, UB)	M ± SD 95% CI (LB, UB)	*p* (intra)	M ± SD 95% CI (LB, UB)	*p* (intra)	M ± SD 95% CI (LB, UB)	*p* (intra)
d_3	33.07 ± 1.81 32.40, 33.75	35.33 ± 1.47 35.33, 1.47	35.15 ± 1.89 34.44, 35.86	2.30 ± 1.63 1.70, 2.91	< 0.001 ***	−0.17 ± 1.89 −0.87, 0.54	1.000 ^NS^	2.14 ± 2.04 1.38, 2.90	< 0.001 ***
d_4	35.03 ± 1.90 34.32, 35.74	37.64 ± 1.95 37.64, 1.95	37.75 ± 1.65 37.13, 38.37	2.61 ± 1.48 2.06, 3.17	< 0.001 ***	0.11 ± 1.59 −0.49, 0.70	1.000 ^NS^	2.72 ± 2.02 1.96, 3.48	< 0.001 ***
d_5	40.64 ± 2.03 39.88, 41.40	42.87 ± 2.14 42.87, 2.14	42.99 ± 1.78 42.32, 43.65	2.23 ± 1.61 1.62, 2.83	< 0.001 ***	0.12 ± 1.47 −0.43, 0.66	1.000 ^NS^	2.34 ± 1.83 1.66, 3.03	< 0.001 ***
d_6	45.59 ± 2.09 44.81, 46.37	48.28 ± 2.69 48.28, 2.69	47.56 ± 2.74 46.54, 48.58	2.69 ± 1.82 2.01, 3.37	< 0.001 ***	−0.72 ± 1.84 −1.40, −0.03	0.123 ^NS^	1.97 ± 2.28 1.12, 2.83	< 0.001 ***
Ratio d_4/d_6	0.77 ± 0.04 0.75, 0.78	0.78 ± 0.04 0.78, 0.04	0.80 ± 0.04 0.78, 0.81	0.01 ± 0.02 0.00, 0.02	0.031 *	0.01 ± 0.04 0.00, 0.03	0.170 ^NS^	0.03 ± 0.05 0.01, 0.04	0.010 *
g_3	25.15 ± 1.90 24.44, 25.86	25.84 ± 1.78 25.84, 1.78	25.22 ± 1.74 24.57, 25.87	0.69 ± 1.40 0.17, 1.21	0.035 *	−0.62 ± 1.23 −1.08, −0.16	0.029 *	0.07 ± 1.87 −0.63, 0.76	1.000 ***
g_4	26.81 ± 2.20 25.98, 27.63	28.58 ± 1.88 28.58, 1.88	28.57 ± 1.43 28.03, 29.11	1.77 ± 1.70 1.13, 2.41	< 0.001 ***	−0.01 ± 1.23 −0.46, 0.45	1.000 ^NS^	1.76 ± 2.04 1.00, 2.52	< 0.001 ***
g_5	30.31 ± 2.11 29.53, 31.10	33.13 ± 2.17 33.13, 2.17	33.47 ± 1.72 32.83, 34.11	2.81 ± 1.61 2.21, 3.42	< 0.001 ***	0.34 ± 1.61 −0.26, 0.94	0.768 ^NS^	3.15 ± 2.13 2.36, 3.95	< 0.001 ***
g_6	32.86 ± 1.77 32.19, 33.52	35.25 ± 2.34 35.25, 2.34	34.83 ± 2.38 33.94, 35.72	2.39 ± 1.66 1.77, 3.01	< 0.001***	−0.42 ± 2.17 −1.23, 0.39	0.902 ^NS^	1.98 ± 2.43 1.07, 2.88	< 0.001 ***
Ratio g_4/g_6	0.82 ± 0.06 0.79, 0.84	0.81 ± 0.05 0.81, 0.05	0.82 ± 0.05 0.80, 0.84	0.00 ± 0.03 −0.02, 0.01	1.000 ^NS^	0.01 ± 0.05 −0.01, 0.03	0.667 ^NS^	0.01 ± 0.06 −0.02, 0.03	1.000 ^NS^
h_3	4.06 ± 1.20 3.61, 4.50	3.98 ± 1.36 3.98, 1.36	4.03 ± 1.44 3.49, 4.56	−0.07 ± 0.86 −0.39, 0.25	1.000 ^NS^	0.04 ± 0.84 −0.27, 0.36	1.000 ^NS^	−0.03 ± 1.25 −0.50, 0.44	1.000 ^NS^
h_4	9.27 ± 1.63 8.67, 9.88	9.54 ± 1.93 9.54, 1.93	9.90 ± 1.69 9.27, 10.53	0.27 ± 1.07 −0.13, 0.67	0.548 ^NS^	0.36 ± 0.76 0.08, 0.65	0.041 *	0.63 ± 1.24 0.17, 1.09	0.028 *
h_5	12.14 ± 1.61 11.54, 12.74	12.77 ± 1.87 12.77, 1.87	13.36 ± 1.75 12.70, 14.01	0.63 ± 0.93 0.28, 098	0.003 **	0.59 ± 0.99 0.22, 0.96	0.009 **	1.22 ± 1.13 0.79, 1.64	< 0.001 ***
h_6	11.48 ± 1.94 10.76, 12.21	12.38 ± 2.04 12.38, 2.04	13.33 ± 2.05 12.56, 14.10	0.90 ± 1.01 0.52, 1.27	< 0.001 ***	0.90 ± 1.01 0.52, 1.27	< 0.001 ***	1.85 ± 1.08 1.44, 2.25	< 0.001 ***
Ratio h_4/h_6	0.82 ± 0.16 0.76, 0.88	0.78 ± 0.18 0.78, 0.18	0.75 ± 0.14 0.70, 0.81	−0.04 ± 0.10 −0.07, 0.00	0.195 ^NS^	−0.03 ± 0.09 −0.06, 0.00	0.186 ^NS^	−0.07 ± 0.09 −0.10, −0.03	0.001 **
APS	343.89 ± 66.04 319.23, 368.55	370.45 ± 63.05 370.45, 63.05	371.58 ± 53.34 351.66, 391.49	26.56 ± 31.92 14.64, 38.48	< 0.001 ***	1.13 ± 38.94 −13.41, 15.67	1.000 ^NS^	27.69 ± 50.03 9.01, 46.37	0.015 *
PPS	1027.98 ± 105.15 988.71, 1067.24	1073.65 ± 115.65 1073.65, 115.65	1071.79 ± 108.93 1031.12, 1112.47	45.67 ± 78.96 16.19, 75.16	0.011 *	−1.86 ± 70.49 −28.18, 24.46	1.000 ^NS^	43.82 ± 75.64 15.57, 72.06	0.011 *
TPS	1371.53 ± 126.95 1324.13, 1418.94	1452.78 ± 123.28 1452.78, 123.28	1443.87 ± 123.36 1397.81, 1489.93	81.25 ± 63.95 57.37, 105.13	< 0.001 ***	−8.91 ± 71.83 −35.73, 17.91	1.000 ^NS^	72.34 ± 104.37 33.36, 111.31	0.002 **

*M* mean, *SD* standard deviation, *CI* confidence intervals and significance levels, *LB* lower bound, *UB* upper bound, *NS* not significant, *PG* patient group, *APS* anterior palatal surface, *PPS* posterior palatal surface, *TPS* total palatal surface

* *p* < 0.05, ** *p* < 0.01, *** *p* < 0.001

Distance measurements in mm, area measurements in mm^2^

**Table 5:** Widths (dental and gingival), heights and areas (anterior, posterior and total) at T1, T2 and T3 in patient group 3, significance *p* intra Breiten (dental und gingival), Höhen und Flächen (anterior, posterior und gesamt) zu den 3 Zeitpunkten T1, T2 und T3 in der Patientengruppe 3, Signifikanzniveau *p* intra

PG3	T1	T2	T3	∆T2-T1		∆T3-T2		∆T3-T1	
Measurement	M ± SD 95% CI (LB, UB)	M ± SD 95% CI (LB, UB)	M ± SD 95% CI (LB, UB)	M ± SD 95% CI (LB, UB)	*p* (intra)	M ± SD 95% CI (LB, UB)	*p* (intra)	M ± SD 95% CI (LB, UB)	*p* (intra)
d_3	34.00 ± 2.24 33.17, 34.84	35.23 ± 2.32 34.36, 36.10	35.24 ± 2.49 34.31, 36.17	1.27 ± 1.56 0.69, 1.86	< 0.001 ***	−0.02 ± 1.11 −0.43, 0.39	1.000 ^NS^	1.25 ± 1.85 0.56, 1.94	0.002 **
d_4	35.38 ± 2.53 34.44, 36.32	37.41 ± 2.64 36.43, 38.40	37.77 ± 2.92 36.68, 38.86	2.03 ± 1.67 1.41, 2.66	< 0.001 ***	0.35 ± 1.88 −0.35, 1.05	0.934 ^NS^	2.39 ± 1.89 1.68, 3.09	< 0.001 ***
d_5	40.69 ± 3.07 39.55, 41.84	42.63 ± 2.95 41.53, 43.74	42.94 ± 3.02 41.81, 44.06	1.94 ± 1.92 1.22, 2.66	< 0.001 ***	0.30 ± 2.38 −0.58, 1.19	1.000 ^NS^	2.24 ± 2.27 1.40, 3.09	< 0.001 ***
d_6	45.66 ± 2.91 44.58, 46.74	48.30 ± 3.14 47.13, 49.48	47.84 ± 3.00 46.72, 48.96	2.64 ± 2.11 1.86, 3.43	< 0.001 ***	−0.46 ± 2.16 −1.27, 0.34	0.747 ^NS^	2.18 ± 1.96 1.45, 2.91	< 0.001 ***
Ratio d_4/d_6	0.78 ± 0.05 0.76, 0.79	0.78 ± 0.04 0.76, 0.79	0.79 ± 0.04 0.78, 0.80	0.00 ± 0.03 −0.01, 0.01	1.000 ^NS^	0.01 ± 0.03 0.00, 0.03	0.050 ^NS^	0.01 ± 0.03 0.00, 0.03	0.112 ^NS^
g_3	25.09 ± 2.17 24.28, 25.90	25.18 ± 1.90 24.47, 25.89	24.76 ± 2.06 23.99, 25.53	0.09 ± 1.32 −0.40, 0.59	1.000 ^NS^	−0.43 ± 0.94 −0.78, −0.07	0.058 ^NS^	−0.33 ± 1.67 −0.96, 0.29	0.846 ^NS^
g_4	26.54 ± 2.38 25.65, 27.43	28.10 ± 2.35 27.22, 28.98	28.46 ± 2.57 27.50, 29.42	1.56 ± 1.51 1.00, 2.13	< 0.001 ***	0.36 ± 1.51 −0.20, 0.92	0.605 ^NS^	1.92 ± 1.75 1.27, 2.58	< 0.001 ***
g_5	30.87 ± 2.83 29.81, 31.93	32.92 ± 2.64 31.94, 33.91	33.20 ± 2.83 32.14, 34.26	2.05 ± 1.39 1.53, 2.57	< 0.001 ***	0.28 ± 2.00 −0.47, 1.03	1.000 ^NS^	2.33 ± 2.30 1.47, 3.19	< 0.001 ***
g_6	32.90 ± 2.72 31.88, 33.91	35.04 ± 2.77 34.00, 36.08	34.64 ± 2.70 33.63, 35.65	2.14 ± 1.80 1.47, 2.82	< 0.001 ***	−0.40 ± 1.73 −1.05, 0.25	0.645 ^NS^	1.74 ± 1.64 1.13, 2.35	< 0.001 ***
Ratio g_4/g_6	0.81 ± 0.06 0.79, 0.83	0.80 ± 0.04 0.79, 0.82	0.82 ± 0.05 0.80, 0.84	−0.01 ± 0.03 −0.02, 0.00	0.874 ^NS^	0.02 ± 0.04 0.00, 0.03	0.029 *	0.01 ± 0.04 0.00, 0.03	0.315 ^NS^
h_3	3.65 ± 1.35 3.15, 4.16	3.99 ± 1.40 3.47, 4.52	4.25 ± 1.77 3.59, 4.91	0.34 ± 0.89 0.01, 0.67	0.133 ^NS^	0.26 ± 0.96 −0.10, 0.62	0.467 ^NS^	0.60 ± 1.13 0.17, 1.02	0.021 *
h_4	9.45 ± 1.89 8.75, 10.16	10.26 ± 2.11 9.48, 11.05	10.67 ± 2.52 9.73, 11.62	0.81 ± 0.71 0.54, 1.08	< 0.001 ***	0.41 ± 0.87 0.09, 0.73	0.044 *	1.22 ± 0.99 0.85, 1.59	< 0.001 ***
h_5	12.90 ± 2.21 12.08, 13.73	13.83 ± 2.52 12.89, 14.77	14.17 ± 2.85 13.11, 15.24	0.93 ± 0.85 0.61, 1.25	< 0.001 ***	0.34 ± 0.84 0.03, 0.65	0.102 ^NS^	1.27 ± 1.08 0.87, 1.67	< 0.001 ***
h_6	12.63 ± 2.24 11.79, 13.46	14.02 ± 2.40 13.12, 14.91	14.74 ± 2.84 13.68, 15.80	1.39 ± 0.87 1.06, 1.72	< 0.001 ***	0.72 ± 1.22 0.27, 1.17	0.009 **	2.98 ± 4.66 1.24, 4.72	< 0.001 ***
Ratio h_4/h_6	0.76 ± 0.12 0.71, 0.80	0.74 ± 0.11 0.70, 0.78	0.73 ± 0.14 0.68, 0.78	−0.02 ± 0.07 −0.05, 0.01	0.387 ^NS^	0.00 ± 0.11 −0.05, 0.04	1.000 ^NS^	−0.02 ± 0.10 −0.06, 0.01	0.540 ^NS^
APS	347.40 ± 73.29 320.03, 374.77	370.87 ± 75.93 342.51, 399.22	384.35 ± 72.18 357.39, 411.30	23.47 ± 47.27 5.82, 41.12	0.033 *	13.48 ± 57.39 −7.95, 34.91	0.625 ^NS^	36.95 ± 68.58 11.34, 62.56	0.019 *
PPS	1084.40 ± 139.54 1032.30, 1136.51	1138.02 ± 126.01 1090.97, 1185.07	1119.58 ± 133.85 1069.60, 1169.56	53.62 ± 65.00 29.35, 77.89	< 0.001 ***	−18.44 ± 70.72 −44.85, 7.97	0.492 ^NS^	35.18 ± 67.38 10.02, 60.34	0.023 *
TPS	1435.14 ± 161.58 1374.80, 1495.47	1508.89 ± 153.90 1451.42, 1566.36	1503.93 ± 175.42 1438.42, 1569.43	73.75 ± 72.07 46.84, 100.66	< 0.001 ***	−4.96 ± 76.33 −33.46, 23.54	1.000 ^NS^	68.79 ± 84.73 37.15, 100.43	< 0.001 ***

*M* mean, *SD* standard deviation, *CI* confidence intervals and significance levels, *LB* lower bound, *UB* upper bound, *NS* not significant, *PG* patient group, *APS* anterior palatal surface, *PPS* posterior palatal surface, *TPS* total palatal surface

* *p* < 0.05, ** *p* < 0.01, *** *p* < 0.001

Distance measurements in mm, area measurements in mm^2^

**Table 6:** Widths (dental and gingival), heights and areas (anterior, posterior and total), significance *p* inter Breiten (dental und gingival), Höhen und Flächen (anterior, posterior und gesamt), Signifikanzniveau *p* inter

	Inter *p*		
Parameter	PG1 vs PG2	PG2 vs PG3	PG3 vs PG1
d_3 ∆T2T1	0.290 ^NS^	0.071 ^NS^	< 0.001 ***
d_3 ∆T3T2	0.975 ^NS^	0.977 ^NS^	0.999 ***
d_3 ∆T3T1	0.191 ^NS^	0.193 ^NS^	0.002 **
d_4 ∆T2T1	0.364 ^NS^	0.412 ^NS^	0.026 *
d_4 ∆T3T2	0.456 ^NS^	0.849 ^NS^	0.194 ^NS^
d_4 ∆T3T1	0.987 ^NS^	0.788 ^NS^	0.697 ^NS^
d_5 ∆T2T1	0.004 **	0.847 ^NS^	< 0.001 ***
d_5 ∆T3T2	0.654 ^NS^	0.935 ^NS^	0.439 ^NS^
d_5 ∆T3T1	0.059 ^NS^	0.982 ^NS^	0.038 *
d_6 ∆T2T1	0.060 ^NS^	0.997 ^NS^	0.050 ^NS^
d_6 ∆T3T2	0.765 ^NS^	0.878 ^NS^	0.976 ^NS^
d_6 ∆T3T1	0.012 *	0.935 ^NS^	0.030 *
Ratio d_4/d_6 ∆T2T1	0.413 ^NS^	0.137 ^NS^	0.791 ^NS^
Ratio d_4/d_6 ∆T3T2	0.092 ^NS^	1.000 ^NS^	0.030 *
Ratio d_4/d_6 ∆T3T1	0.020 *	0.546 ^NS^	0.203 ^NS^
g_3 ∆T2T1	< 0.001 ***	0.263 ^NS^	< 0.001 ***
g_3 ∆T3T2	0.713 ^NS^	0.816 ^NS^	0.983 ^NS^
g_3 ∆T3T1	< 0.001 ***	0.669 ^NS^	< 0.001 ***
g_4 ∆T2T1	0.050 ^NS^	0.900 ^NS^	0.016 *
g_4 ∆T3T2	0.106 ^NS^	0.621 ^NS^	0.010 *
g_4 ∆T3T1	0.801 ^NS^	0.946 ^NS^	0.946 ^NS^
g_5 ∆T2T1	0.099 ^NS^	0.207 ^NS^	< 0.001 ***
g_5 ∆T3T2	0.962 ^NS^	0.992 ^NS^	0.988 ^NS^
g_5 ∆T3T1	0.350 ^NS^	0.326 ^NS^	0.016 *
g_6 ∆T2T1	0.058 ^NS^	0.879 ^NS^	0.017 *
g_6 ∆T3T2	0.985 ^NS^	0.999 ^NS^	0.991 ^NS^
g_6 ∆T3T1	0.058 ^NS^	0.907 ^NS^	0.020 *
Ratio g_4/g_6 ∆T2T1	0.962 ^NS^	0.985 ^NS^	0.904 ^NS^
Ratio g_4/g_6 ∆T3T2	0.043 *	0.693 ^NS^	0.004 **
Ratio g_4/g_6 ∆T3T1	0.225 ^NS^	0.940 ^NS^	0.013 *
h_3 ∆T2T1	0.030 *	0.165 ^NS^	0.732 ^NS^
h_3 ∆T3T2	0.721 ^NS^	0.621 ^NS^	0.209 ^NS^
h_3 ∆T3T1	0.331 ^NS^	0.077 ^NS^	0.721 ^NS^
h_4 ∆T2T1	0.170 ^NS^	0.049 *	0.836 ^NS^
h_4 ∆T3T2	0.085 ^NS^	0.978 ^NS^	0.054 ^NS^
h_4 ∆T3T1	0.950 ^NS^	0.116 ^NS^	0.059 ^NS^
h_5 ∆T2T1	0.797 ^NS^	0.460 ^NS^	0.846 ^NS^
h_5 ∆T3T2	0.988 ^NS^	0.632 ^NS^	0.540 ^NS^
h_5 ∆T3T1	0.759 ^NS^	0.981 ^NS^	0.860 ^NS^
h_6 ∆T2T1	0.980 ^NS^	0.183 ^NS^	0.257 ^NS^
h_6 ∆T3T2	0.532 ^NS^	0.752 ^NS^	0.177 ^NS^
h_6 ∆T3T1	0.862 ^NS^	0.302 ^NS^	0.598 ^NS^
Ratio h_4/h_6 ∆T2T1	0.512 ^NS^	0.820 ^NS^	0.869 ^NS^
Ratio h_4/h_6 ∆T3T2	0.033 *	0.572 ^NS^	0.002 **
Ratio h_4/h_6 ∆T3T1	0.509 ^NS^	0.254 ^NS^	0.033 *
APS ∆T2T1	0.216 ^NS^	0.952 ^NS^	0.122 ^NS^
APS ∆T3T2	0.999 ^NS^	0.704 ^NS^	0.623 ^NS^
APS ∆T3T1	0.506 ^NS^	0.796 ^NS^	0.884 ^NS^
PPS ∆T2T1	0.228 ^NS^	0.901 ^NS^	0.444 ^NS^
PPS ∆T3T2	0.983 ^NS^	0.598 ^NS^	0.709 ^NS^
PPS ∆T3T1	0.350 ^NS^	0.898 ^NS^	0.165 ^NS^
TPS ∆T2T1	0.122 ^NS^	0.922 ^NS^	0.052 ^NS^
TPS ∆T3T2	0.978 ^NS^	0.977 ^NS^	1.000 ^NS^
TPS ∆T3T1	0.232 ^NS^	0.990 ^NS^	0.181 ^NS^

*M* mean, *SD* standard deviation, *CI* confidence intervals and significance levels, *NS* not significant, *PG* patient group, *APS* anterior palatal surface, *PPS* posterior palatal surface, *TPS* total palatal surface

* *p* < 0.05, ** *p* < 0.01, *** *p* < 0.001

Distance measurements in mm, area measurements in mm^2^

### Patients and treatment duration

The average age at insertion and first activation of the appliance was 10.7 ± 2.17 years, with the youngest patient being 5.6 years old and the oldest 15.7 years old.

In PG 1 (*n* = 30, < 10 years of age), 13 girls (43.3%) and 17 boys (56.7%) were between 5.6 and 9.5 years of age. The time interval between T1 and T2 was 17.0 ± 8.4 months; the interval between T2 and T3 was 19.5 ± 14.3 months.

In PG2 (*n* = 30, 10–< 13 years of age) there were 18 girls (60.0%) and 12 boys (40%) aged between 10.0 and 11.9 years. The interval between T1 and T2 in this group was 16.8 ± 7.0 months and the interval between T2 and T3 was 20.8 ± 9.5 months.

PG3 (*n* = 30, ≥ 13 years of age) consisted of 15 girls (50.0%) and 15 boys (50%) aged between 12.0 and 15.7 years. The delta between T1 and T2 in this group was 16.6 ± 7.0 months and the delta between T2 and T3 was 20.5 ± 10.6 months.

### Width (dental, gingival)

#### Intragroup transverse changes

In all three groups, the palatal width increased markedly during the treatment period ∆T1–T2 at the dental and gingival levels on all measuring sections (except PG3 g_3). The amounts were greater at the dental level than at the gingival level in all treatment groups apart from the measurement sections on the 5th and 6th teeth in PG2 and the 5th teeth in PG3. During the second treatment phase (∆T2–T3), the widths did not change except for the distance g_3 in PG 2. As a long-term effect (∆T1–T3), the increases in width at the dental and gingival levels remained stable in all measurement sections except for g_3 in PG2 and PG3.

#### Intergroup transverse changes

The palate width increases differed noticeably between the groups at the following points and treatment stages:

Short-term changes of plate appliance treatment (∆T1–T2): PG1 vs PG2 at d_5 and g_3, PG1 vs PG3 at d_3, d_4, d_5, g_3, g_4, g_5, g_6, whereby basically all values in PG1 were greater than in PG2, and in PG2 again greater than in PG3.

Short-term changes during second treatment phase (∆T2–T3): PG1 vs PG3 at g_4.

Long-term changes (∆T1–T3): PG1 vs PG2 on d_6 and g_3, PG1 vs PG3 on d_3, d_5, d_6, g_3, g_5, g_6.

#### Anterior to posterior width ratio within and between PG1, 2 and 3

In all three groups, the values at T1, T2 and T3 were <1 at both the dental and gingival levels. This describes a relatively larger width increase in the posterior region and thus an inverse V‑shaped transverse expansion. Within the groups, the extent of the changes differed noticeably between the following intervals: PG1 gingival ∆T3–T1, PG2 dental ∆T2–T1 and ∆T3–T1, PG3 gingival ∆T3–T2. Between the three groups, the changes at both measurement levels were not remarkable at any time.

### Palatal vault height

#### Height changes within patient groups

The palatal vault height increased markedly during the treatment period ∆T1–T2 apart from PG2 h_3 and h_4 as well as PG3 h_3.

The values increased from anterior to posterior in all patient groups. During the second treatment phase (∆T2–T3), the heights changed clearly in PG1 at the measurement sections h_5 and h_6, in PG2 at h_4, h_5 and h_6 and in PG3 at h_4 and h_6. As a long-term effect (∆T1–T3), distinct height increases occurred except for h_3 and h_4 in PG1 and h_3 in PG2.

#### Height changes between patient groups

The changes in height differed clearly between the groups during the treatment period ∆T1–T2 at the following points: PG1 vs PG2 at h_3, PG1 vs PG3 at h_4, whereby all amounts in PG2 were generally smaller than in PG1 and PG3, except for h_3 being larger in PG3 than in PG1.

There were no distinct differences in both ∆T2–T3 and ∆T1–T3.

#### Anterior to posterior height ratio within and between PG1, 2 and 3

All patient groups showed height ratio values of < 1 at T1, T2 and T3. This describes a relatively greater increase in height in the posterior region in the sagittal plane. Within the groups, the extent of the changes differed remarkably between the following time points: PG1 ∆T3–T2 and ∆T3–T1, and PG2 ∆T3–T1. Between all groups, the changes at both measurement levels were not remarkable at any time.

### Changes of palate surface

#### Intragroup comparison

The palate surface (anterior, posterior, total) increased significantly during the treatment period ∆T1–T2 in all groups. With one exception (anterior palate PG2 vs PG 3), the increases were greater in PG1 than in PG2 and again greater in PG2 than in PG3, although the initial anatomical volume was lower in each case. During the second treatment phase (∆T2–T3), the surfaces did not change noticeably. As a long-term effect (∆T1–T3), the extent of palatal changes was perceptible in all three groups.

#### Intergroup comparison

The intergroup comparison showed no remarkable differences for the increase in anterior, posterior and total palatal area.

## Discussion

The plate appliance as a removable single-jaw appliance originally goes back to descriptions by Nord [16] and Schwarz [17] and is widely used today [18]. One advantage of the plate appliance is its unrestricted hygiene capability, which can be particularly advantageous when choosing a treatment appliance for patients with high caries activity [19].

Removable appliances can be successfully used to correct malocclusions according to the standards of the Peer Assessment Rating (PAR) index [20]. In addition to the indication and design, the prerequisites for successful treatment are good patient cooperation and very good specialist knowledge on the part of the practitioner [19, 20].

If the maxillary dental arch is constricted, a plate appliance with a transverse screw allows arch expansion until congruence with the mandibular dental arch is achieved. The screw is generally the best active element of a plate appliance to control [21]. It should be incorporated in the centre of the plate body [19] so that the anterior and posterior teeth are moved equally buccally by the rigid plate body when activated [22]. This activation of the screw thread should be carried out depending on the periodontal gap so that the alveolar bone can be remodelled in a targeted manner and within its physiological range [21]. The course of treatment can be significantly influenced by the activation intervals. The prescription of optimal activation is therefore an essential component of the treatment plan. An individual activation interval selected for the respective type of expansion screw can help to optimise treatment [23].

In the present study, an identical appliance construction including a central transverse expansion screw was used in all patients. In addition, the specified wearing time and the activation intervals of the screw spindle were the same for all patients in any group. To our knowledge, there are currently no studies describing a standardised expansion protocol. A recent study by Van de Velde et al. [24] also investigated interceptive expansion treatment with removable plate appliances in a study group of the same size. However, they partially used nonstandardized appliances addressing additional tooth movement. Furthermore, they did not differentiate patient age at treatment begin, which has proven to be very important for outcome quality in expansion protocols [10].

When comparing the therapeutic effects of the plate appliance treatment, it is noticeable that the increases in width, height and surface measurements were greatest in PG1 and smallest in PG3. On the one hand, this was because the initial values were also lowest in PG1 and highest in PG3 for age-related anatomical reasons and, on the other hand, screw activation was prescribed for all patients until the therapeutic goal (crossbite correction, achievement of dental arch congruence) was achieved.

Regarding the therapeutic effects, it is also noticeable that the width increase in the posterior region was greater than in the anterior region in all patient groups and that the palatal height increased from anterior to posterior. Weyrich et al. [25] obtained analogous results for width measurements in the transverse plane. They measured the casts of 10 patients who were treated with a maxillary plate appliance with an incorporated transverse screw as part of a comparative study with two different RME appliances: the transverse width measured dentally increased more in the first permanent molar than that in the deciduous molar or premolar region.

This indicates that force transmission through plate appliances is particularly efficient in the posterior region. In this context, the results of a finite element method study by Geramy and Shahroudi [25] are of interest: they found that during palatal expansion in the first permanent molar region, the degree of buccal tilt, the crestal and apical stress levels, the total stress in the periodontium of the anchor teeth and the stress in both the cortical and spongy bone were even higher with removable appliances than with fixed appliances.

Tai and Park [27] and Tai et al. [28] were able to show in CBCT studies that plate appliances in the maxilla preferentially influenced the dentoalveolar complex and that there were varying effects on the alveolar width: the amounts of the increases decreased both palatally and buccally measured from the enamel–cement junction towards the apical direction. In the present study, this corresponds to the different changes in width at the dental and gingival levels.

Tai et al. [28] observed a change in the maxillary bone width, but not in the mandibular bone, because of treatment with plate appliances. Tai and Park [27] were even able to demonstrate separation of the midpalatal sutures in individual patients. They concluded that plate appliances can generate considerable forces that can lead to stimulation of the midpalatal suture, especially in younger patients, provided that the appliance is worn for many hours.

According to Tränkmann [21], the choice of treatment timing is also important, as only expected jaw growth, but not already completed jaw growth, can be influenced. The aim should be to achieve dental arch congruence as early as possible so that the further development of the dentition can be stimulated by the occlusion.

Nevertheless, the present study showed that the increases in width, height and surface differed only slightly between the patient groups and did not relapse: the morphological changes were comparable in patients of different ages, with the greatest effects being observed in the posterior molar region. Early, main and late treatments were equally effective. This may be because the treatment effects with plate appliances are predominantly attributable to dentoalveolar effects and effects on the sutures only play a subordinate role. The results were also stable at the time of follow-up and did not relapse. This could be due to the quality of further treatment and retention.

## Study limitations

Any study that relies on convenience sampling for retrospective investigations will provide results with limited generalisability due to selection bias. This was mitigated by strict adherence to a single appliance design. Another probable limitation is the lack of an untreated control group. The consequence is that the determined values must be regarded as the sum from natural growth and therapeutic effects. However, it should be regarded that the amount of natural transverse jaw growth in adolescents is low [29, 30]. Previous studies of plate appliance treatment that included a control group showed significant differences between the treatment effect and the changes brought about by growth of the control group, suggesting that the changes were primarily due to orthodontic treatment [28, 29, 31] and not to the effects of natural growth.

## Conclusions

Transverse expansion of the upper jaw with plate appliances led to a significant width increase at the tooth and gingival level. The width increase was greater posteriorly than anteriorly, regardless of the age of the patient at the start of treatment. The palatal height also increased significantly from anterior to posterior during treatment in almost all measured sections in all observed groups. Overall, the treatment-related changes were more pronounced in width than in height. The palatal surface measurements also showed increases in all groups after the plate appliance treatment. The changes in width, height and surface area remained stable during the investigation period.

The morphological changes with plate appliances were comparable in patients of different ages. Early, main and late treatments were equally effective. This is probably because the effects of plate appliance treatment are mainly due to dentoalveolar effects, with effects on the sutures playing a minor role.

## Data Availability

The data supporting the findings of this research can be obtained directly from the authors of the study.

## References

[1] Saghiri MA, Eid J, Tang CK, Freag P (2021) Factors influencing different types of malocclusion and arch form—a review. J Stomatol Oral Maxillofac Surg 122:185–191 32659411 10.1016/j.jormas.2020.07.002

[2] Švalkauskienė V, Šmigelskas K, Šalomskienė L, Andriuškevičiūtė I, Šalomskienė A, Vasiliauskas A, Šidlauskas A (2015) Heritability estimates of dental arch parameters in lithuanian twins. Stomatologija 17:3–8 26183851

[3] Al-Zubair NM (2015) Determinant factors of yemeni maxillary arch dimensions. Saudi Dent J 27:50–54 25544815 10.1016/j.sdentj.2014.08.005 PMC4273290

[4] Alkadhi OH, Almahfouz SF, Tokhtah HA, Binhuwaishel LA (2018) Dental arch dimensions in saudi adults. Int J Dent: 2190250 10.1155/2018/2190250 PMC5832096 29666645

[5] Aznar T, Galán AF, Marín I, Domínguez A (2006) Dental arch diameters and relationships to oral habits. Angle Orthod 76:441–445 16637724 10.1043/0003-3219(2006)076[0441:DADART]2.0.CO;2

[6] Herrera S, Pierrat V, Kaminski M, Benhammou V, Marchand-Martin L, Morgan AS, Le Norcy E, Ancel P‑Y, Germa A (2022) Risk factors for high-arched palate and posterior crossbite at the age of 5 in children born very preterm: EPIPAGE‑2 cohort study. Front Pediatr 10:784911 35498807 10.3389/fped.2022.784911 PMC9051072

[7] Lindner A, Modéer T (1989) Relation between sucking habits and dental characteristics in preschoolchildren with unilateral cross-bite. Scand J Dent Res 97:278–283 2740839 10.1111/j.1600-0722.1989.tb01613.x

[8] Modéer T, Odenrick L, Lindner A (1982) Sucking habits and their relation to posterior cross-bite in 4‑year-old children. Scand J Dent Res 90:323–328 6957971 10.1111/j.1600-0722.1982.tb00744.x

[9] Lione R, Buongiorno M, Franchi L, Cozza P (2014) Evaluation of maxillary arch dimensions and palatal morphology in mouth-breathing children by using digital dental casts. Int J Pediatr Otorhinolaryngol 78:91–95 24300946 10.1016/j.ijporl.2013.09.028

[10] Kinzinger GSM, Lisson JA, Buschhoff C, Hourfar J, Korbmacher-Steiner H (2022) Impact of rapid maxillary expansion on palatal morphology at different dentition stages. Clin Oral Investig 26:4715–4725 35267098 10.1007/s00784-022-04434-9 PMC9276570

[11] Kinzinger GSM, Hourfar J, Buschhoff C, Heller F, Korbmacher-Steiner HM, Lisson JA (2022) Age-dependent interactions of maxillary sutures during RME and their effects on palatal morphology: CBCT and dental cast analysis. J Orofac Orthop 83:412–431 36205766 10.1007/s00056-022-00429-z PMC9596587

[12] Kinzinger GSM, Lisson JA, Buschhoff C, Hourfar J (2023) Age-dependent effects on palate volume and morphology during orthodontic RME treatment. Clin Oral Investig 27:2641–2652 36602590 10.1007/s00784-022-04831-0 PMC10264469

[13] Pont A (1909) Der Zahnindex in der Orthodontie. Z Zahnärztl Orthop 3:306–315

[14] Gracco A, Malaguti A, Lombardo L, Mazzoli A, Raffaeli R (2010) Palatal volume following rapid maxillary expansion in mixed dentition. Angle Orthod 80:153–159 19852655 10.2319/010407-7.1 PMC8978743

[15] Dahlberg G (1940) Statistical methods for medical and biological students. Br Med J 2:358–359

[16] Nord CF (1928) Loose appliances of orthodontia. Dent. Cosmos 70:681

[17] Schwarz AM (ed) (1938) Gebissregelung mit Platten. Urban & Schwarzenberg, Wien

[18] Sandstrom RA, Klapper L, Papaconstantinou S (1988) Expansion of the lower arch concurrent with rapid maxillary expansion. Am J Orthod Dentofacial Orthop 94:296–302 3052025 10.1016/0889-5406(88)90054-6

[19] Lisson JA (2022) Kieferorthopädische Behandlung mit Plattenapparaturen – Möglichkeiten und Grenzen. Quintessenz Zahnmed 73:938–947

[20] Miethke RR, Wronski C (2009) What can be achieved with removable orthodontic appliances? J Orofac Orthop 70:185–199 19484412 10.1007/s00056-009-0818-x

[21] Tränkmann J (1996) Die aktualisierte Plattenapparatur in der Kieferorthopädie. The modified removable appliance in facial orthopaedics. Kieferorthop 10:95–110

[22] Tränkmann J (1993) Leistungsfähigkeit modifizierter Plattenapparaturen. The efficiency of modified removable plate appliances. Prakt Kieferorthop 7:9–22

[23] Schott TC, Engelhard L, Gómez-Serrano D, Meyer-Gutknecht H (2011) Comparison of estimated and actual changes in gap widths of expansion screws in plate appliances with 7‑ and 14-day activation. J Orofac Orthop 72:446–456 22124509 10.1007/s00056-011-0049-9

[24] Van de Velde AS, De L B, de Cadenas Llano-Pérula M, Laenen A, G W (2024) Long-term effects of orthodontic interceptive expansion treatment: a retrospective study. J Orofac Orthop 85:371–380 37115290 10.1007/s00056-023-00467-1

[25] Weyrich C, Noss M, Lisson JA (2010) Comparison of a modified RME appliance with other appliances for transverse maxillary expansion. J Orofac Orthop 71:265–272 20676813 10.1007/s00056-010-9945-7

[26] Geramy A, Shahroudi AS (2014) Fixed versus removable appliance for palatal expansion; A 3D analysis using the finite element method. J Dent 11:75–84 PMC4037269 24910679

[27] Tai K, Park JH (2010) Dental and skeletal changes in the upper and lower jaws after treatment with schwarz appliances using cone-beam computed tomography. J Clin Pediatr Dent 35:111–120 21189775 10.17796/jcpd.35.1.b21t44rj34027603

[28] Tai K, Park JH, Mishima K, Shin J‑W (2011) 3‑Dimensional cone-beam computed tomography analysis of transverse changes with Schwarz appliances on both jaws. Angle Orthod 81:670–677 21406000 10.2319/110910-655.1 PMC8919744

[29] Erdinç AE, Ugur T, Erbay E (1999) A comparison of different treatment techniques for posterior crossbite in the mixed dentition. Am J Orthod Dentofacial Orthop 116:287–300 10474101 10.1016/s0889-5406(99)70240-4

[30] Thilander B (2009) Dentoalveolar development in subjects with normal occlusion. A longitudinal study between the ages of 5 and 31 years. Eur J Orthod 31:109–120 19304760 10.1093/ejo/cjn124

[31] Pałka J, Gawda J, Byś A, Zawadka M, Gawda P (2022) Assessment of growth changes in the width of dental arches caused by removable appliances over a period of 10 months in children with malocclusion. Int J Environ Res Public Health 19:3442 35329130 10.3390/ijerph19063442 PMC8950693

